# Characterization of Volatile Organic Compounds in Five Celery (*Apium graveolens* L.) Cultivars with Different Petiole Colors by HS-SPME-GC-MS

**DOI:** 10.3390/ijms241713343

**Published:** 2023-08-28

**Authors:** Yue Sun, Mengyao Li, Xiaoyan Li, Jiageng Du, Weilong Li, Yuanxiu Lin, Yunting Zhang, Yan Wang, Wen He, Qing Chen, Yong Zhang, Xiaorong Wang, Ya Luo, Aisheng Xiong, Haoru Tang

**Affiliations:** 1 College of Horticulture, Sichuan Agricultural University, Chengdu 611130, China; serenity180@163.com (Y.S.); limy@sicau.edu.cn (M.L.); lxy2324804342@163.com (X.L.); jiageng_du@163.com (J.D.); 15865899720@163.com (W.L.); linyx@sicau.edu.cn (Y.L.); asyunting@sicau.edu.cn (Y.Z.); wangyanwxy@sicau.edu.cn (Y.W.); hewen0724@gmail.com (W.H.); supnovel@sicau.edu.cn (Q.C.); zhyong@sicau.edu.cn (Y.Z.); wangxr@sicau.edu.cn (X.W.); luoya945@sicau.edu.cn (Y.L.); 2 College of Horticulture, Nanjing Agricultural University, Nanjing 210095, China; xiongaisheng@njau.edu.cn

**Keywords:** HS-SPME-GC-MS, stoichiometry, volatile organic compounds, flavor quality, celery

## Abstract

Celery (*Apium graveolens* L.) is an important vegetable crop cultivated worldwide for its medicinal properties and distinctive flavor. Volatile organic compound (VOC) analysis is a valuable tool for the identification and classification of species. Currently, less research has been conducted on aroma compounds in different celery varieties and colors. In this study, five different colored celery were quantitatively analyzed for VOCs using HS-SPME, GC-MS determination, and stoichiometry methods. The result revealed that γ-terpinene, d-limonene, 2-hexenal,-(E)-, and β-myrcene contributed primarily to the celery aroma. The composition of compounds in celery exhibited a correlation not only with the color of the variety, with green celery displaying a higher concentration compared with other varieties, but also with the specific organ, whereby the content and distribution of volatile compounds were primarily influenced by the leaf rather than the petiole. Seven key genes influencing terpenoid synthesis were screened to detect expression levels. Most of the genes exhibited higher expression in leaves than petioles. In addition, some genes, particularly *AgDXS* and *AgIDI*, have higher expression levels in celery than other genes, thereby influencing the regulation of terpenoid synthesis through the MEP and MVA pathways, such as hydrocarbon monoterpenes. This study identified the characteristics of flavor compounds and key aroma components in different colored celery varieties and explored key genes involved in the regulation of terpenoid synthesis, laying a theoretical foundation for understanding flavor chemistry and improving its quality.

## 1. Introduction

Celery (*Apium graveolens* L.) is one of the important Apiaceae family crops; it is native to the Mediterranean and Middle Eastern regions but is now cultivated worldwide [1]. Celery is not only a vegetable but also a vital raw material for cosmetics and spices. Its extracts have several medicinal properties, including antibacterial, anti-inflammatory, hypoglycemic, and hypolipidemic effects [2]. The leaves and petioles are the main edible parts of celery, with the petioles displaying a range of colors including white, yellow, green, or purple. Celery is full of bioactive compounds, including flavonoids, phenolic acids, furocoumarins, terpenoids, and phthalides [3,4]. Celery’s unique and full-bodied flavor and aroma are affected by varieties, seasons, geographic location, and other factors. The fragrance of different celery varieties differs noticeably [5]. Aromatic compounds are the crucial factors influencing the flavor quality of fresh and processed vegetables and fruits [6]. As plants grow and develop, the volatile organic compound (VOC) content changes, imparting celery with its distinctive aromatic characteristics, which have an important impact on its sensory quality [7]. VOCs are low-boiling and volatile small molecule compounds produced by secondary metabolic pathways [8], which benefit mutual interactions between plants and the environment to improve autologous competitive ability via inhibiting other plants, including attracting pollinating insects and seed dispersers and resisting parasites, pathogens, and herbivores [9]. VOCs can be biosynthesized by all organs in plants, including roots, stems, leaves, flowers, fruits, and seeds [10]. The VOCs can be classified into esters, phenols, alcohols, amino acids, and terpenes based on their biosynthesis pathways [11]. HS-SPME-GC-MS analysis of celery extracts reveals that 70% of VOCs are terpenes, with D-limonene being the most significant. The aroma of celery seeds is derived from phthalide, while terpenoids are the primary VOCs in the essential oil of celery seeds. During the five development stages of celery seed, the levels of 85 VOCs vary significantly, including 20 terpenoids and six phthalides [12]. The aroma characteristics of celery are determined by factors; for example, Turner discovered harvest years contribute to significant differences in the volatile components and sensory profile of celery, as samples harvested in 2018 contained higher proportions of sesquiterpenes due to higher temperatures [5]. VOC analysis can authenticate and classify varieties; machine olfaction, based on electronic-nose (e-nose) technologies and VOC emissions from plant parts, is readily available for rapid identification of fruit and vegetative agricultural products, especially grape cultivar identification [13]. 

Traditional methods conclude solvent extraction and steam distillation for the enrichment and analysis of plant VOCs, whereas modern extraction methods conclude Headspace-Solid Phase Micro Extraction (HS-SPME), enzyme-assisted extraction, and microwave-assisted extraction [14]. The combination of HS-SPME and Gas Chromatography-Mass Spectrometry (GC-MS) is a well-established method for analyzing fruit aroma [15]. The HS-SPME method is simple to operate and requires minimal equipment. Compared with solvent extraction, it has the advantages of sampling, enrichment, and injection in a single unit, being fast and simple to operate, and requiring no solvent [16]. It is a widely used and environmentally friendly method for extracting volatile and semi-volatile organic substances with excellent aroma reduction and no organic reagent interference [17]. HS-SPME and GC-MS were used to enrich and analyze volatiles in ’Eureka’ and ‘Xiangshui’ lemon pulps, which had higher proportions of alcohols, aldehydes, and esters 186 days after flowering [18]. VOCs of *Chimonanthus praecox* flowers were analyzed using optimized SPME conditions, and three potential biomarker compounds (n-pentadecane, n-cetane, and n-heptadecane) were found in the living and excised flowers [19].

An extensive body of literature reports that many genes are involved in the VOC synthesis pathway and regulate the accumulation of volatiles. Several types of genes, including *WRKY*, *MYB*, and *ERF*/*AP2,* have been implicated in the direct or indirect regulation of VOCs in horticultural plants [20,21]. *FaMYB63* can positively regulate eugenol production by activating transcription of the regulatory genes *FaEOBII* and *FaMYB10* and the genes encoding eugenol biosynthesis enzymes *FaEGS1*, *FaEGS2*, and *FaCAD1* during early and late strawberry fruit development [22]. The terpene synthesis pathway is categorized into the mevalonate (MVA) and methylerythritol phosphate (MEP) pathways. The MVA pathway occurs within the cytoplasm, whereas the MEP pathway takes place in the plastid [23]. The key rate-limiting enzyme of the MEP pathway is the extensively studied 1-deoxy-D-xylulose-5-phosphate synthase (*DXS*) [24]. 1-deoxy-d-xylulose 5-phosphate reductoisomerase (*DXR*) is also an important enzyme involved in terpenoid biosynthesis via the MEP plastid pathway and is responsible for catalyzing the second step of terpenoid biosynthesis in the chloroplast [25]. Whereas in the MVA pathway, the key rate-limiting enzyme is hydroxymethylglutaryl-CoA reductase (*HMGR*) [26]. Overexpression of *PtHMGR* modifies the MVA- and MEP-related genes expression, increasing the abscisic acid (ABA), gibberellic acid (GA), carotene, and lycopene content [27]. Two types of isopentenyl diphosphate dimethylallyl diphosphate isomerase (*IDI-1* and *IDI-2*) catalyze the interconversion of isopentenyl diphosphate (IPP) and dimethylallyl diphosphate (DMAPP), which are the structural foundation for isoprenoid compound biosynthesis [28]. Geranyl pyrophosphate synthase (*GPS*), farnesyl pyrophosphate synthase (FPPS), and geranylgeranyl pyrophosphate synthase (*GGPS*) are precursor genes for the biosynthesis of terpenoids, including zeatins, monoterpenoids, sesquiterpenoids, diterpenoids, triterpenoids, and steroids [29]. Terpenes are essential secondary metabolites for celery, determining its flavor. Li et al. conducted a study that identified the presence of *AgTPS* in specific celery tissues, which were found to be unevenly distributed [30]. 

Currently, the research on celery VOCs is not deep enough to identify varieties, and the correlation between various cultivars and their respective colors is seldom investigated. In this research, five different colored celery varieties were selected, and HS-SPME-GC-MS was used to extract VOCs from celery. To gain a better understanding of the distribution patterns of volatile organic compounds (VOCs) in celery, we compared and analyzed the volatile fractions and conducted correlation studies on the leaves and petioles of different celery varieties and colors. In this study, a comprehensive study was conducted that integrated the unique aroma of celery with an examination of prevailing aroma preferences, thereby establishing a framework for evaluating the processing and quality of celery. By analyzing the characteristic flavor compounds present in different celery samples, we identified the essential aroma constituents of various celery varieties, thereby laying the groundwork for enhancing the overall flavor quality of celery. 

## 2. Results

### 2.1. Total Ion Flow Diagram of Celery VOCs

**Figure 1:**
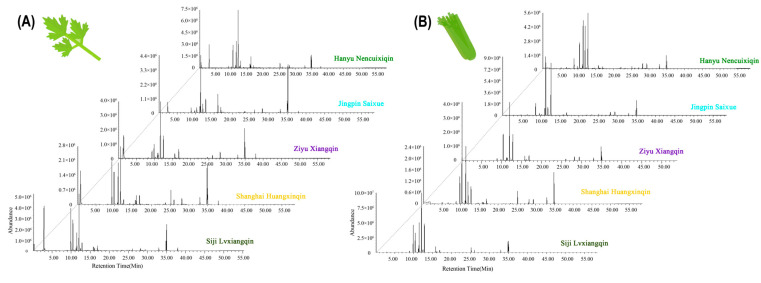
Total ion flow diagram of volatile compounds in leaves (**A**) and petioles (**B**) of five celery varieties. Different colors of writing screened colors of celery, including green for ‘Hanyu Nencuixiqin’ and ‘Siji Lvxiangqin’, white for ‘Jingpin Saixue’, purple for ‘Ziyu Xiangqin’, and yellow for ‘Shanghai Huangxinqin’.

As shown in Figure 1, the total ion flow diagrams of VOCs for five varieties of celery leaves and petioles differ significantly. Substances were analyzed qualitatively and quantitatively using the VOC database, characteristic ion fragments, and concentrations of internal standard substances. For all celery varieties, all compound contents in leaves were above 10^6^, with *A. graveolens* ‘Hanyu Nencuixiqin’ and ‘Siji LvxiangqinLvxiangqin’ (green celery for (E)-β-ocimene at the RT of 12.1th) having the highest peak (Figure 1A). Figure 1B shows that the petioles of ‘Siji LvxiangqinLvxiangqin’ (green celery) and ‘Jingpin Saixue’ (white celery) had the highest peak. The retention times (RT) of the 10th to 13th and 35th min were the most intense. Although peak areas, heights, and RT varied among species, the overall trend was consistent, and celery’s characteristic compounds peaked during these two time periods.

### 2.2. Composition and Content of VOCs

The leaves and petioles of five celery varieties were analyzed for 183 VOCs, including 60 terpenes, 34 aromatic compounds, 21 esters, 21 alcohols, 19 alkanes, 12 heterocyclic compounds, 10 polymeric aldehydes, and 6 aldehydes (Table 1 and Table S1).

**Figure 2:**
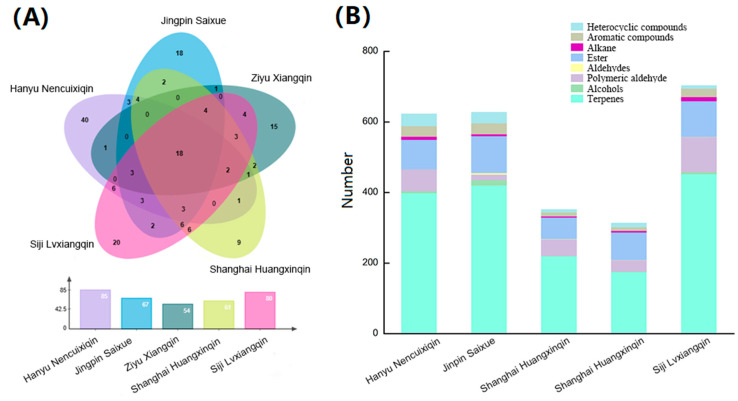
Analysis of compound species of five celery varieties. (**A**) A Venn diagram showed the differences and common compounds of five celery varieties. (**B**) The percentage stacking chart of eight kinds of compounds in five celery varieties.

*A. graveolens* ‘Hanyu Nencuixiqin’ had 85 compounds, followed by ‘Siji Lvxiangqin’, ‘Jingpin Saixue’, ‘Shanghai Huangxinqin’, and ‘Ziyu Xiangqin’, with 80, 67, 61, and 54, respectively (Figure 2). Different colors of celery contained compounds and constituents that varied significantly. Green celery contains more VOC species than white, yellow, and purple celery, with the following contents in descending order: ‘Siji Lvxiangqin’ (green), ‘Jingpin Saixue’ (white), ‘Hanyu Nencuixiqin’ (green), ‘Shanghai Huangxinqin’ (yellow), and ‘Ziyu Xiangqin’ (purple). ‘Hanyu Nencuixiqin’ and ‘Siji Lvxiangqin’ contained 40 and 20 characteristic compounds, respectively, while ‘Shanghai Huangxinqin’ contained the least. The five varieties mutually shared eighteen volatile compounds, including nine terpenes: neo-allo-ocimene, 5-pentylcyclohexa-1,3-diene, β-myrcene, γ-terpinene, d-limonene, (E)-β-ocimene, caryophyllene, β-ocimene, neophytadiene; four alkanes: nonane cyclomethicone 5, cyclohexasiloxane,-dodecamethyl-, cyclomethicone 7; one aromatic compounds: naphthalene; two esters: neocnidilide, senkyunolide; one polymeric aldehyde: 2-hexenal,-(E)-; and one heterocyclic compounds, kessane.

As shown in Table 2, terpenes had the highest VOC content in all varieties and tissues, followed by polymeric aldehydes, esters, heterocyclic compounds, aromatic compounds, alkanes, alcohols, and aldehydes. All celery varieties had the highest content of terpenes, followed by esters, and the lowest content of aldehydes. In ‘Hanyu Nencuixiqin’, ‘Jingpin Saixue’, ‘Ziyu Xiangqin’, ‘Shanghai Huangxinqin’, and ‘Siji Lvxiangqin’, the terpenes content (µg/g) were 398.60, 407.76, 220.20, 150.36, 452.43, ester content (µg/g) 76.81, 104.45, 60.44, 77.57, 101.02, and aldehydes content (µg/g) 0.43, 4.60, 0.66, 0.45, 0.36, respectively. Most of the varieties, such as ‘Hanyu Nencuixiqin’, ‘Ziyu Xiangqin’, and ‘Shanghai Huangxinqin’, contained more compounds in the leaf than in the petiole, whereas ‘Jingpin Saixue’ and ‘Siji Lvxiangqin’ contained more compounds in the petioles than in the leaves.

### 2.3. Comparison of Leaf Blade Petiole VOC Differentiation

**Figure 3:**
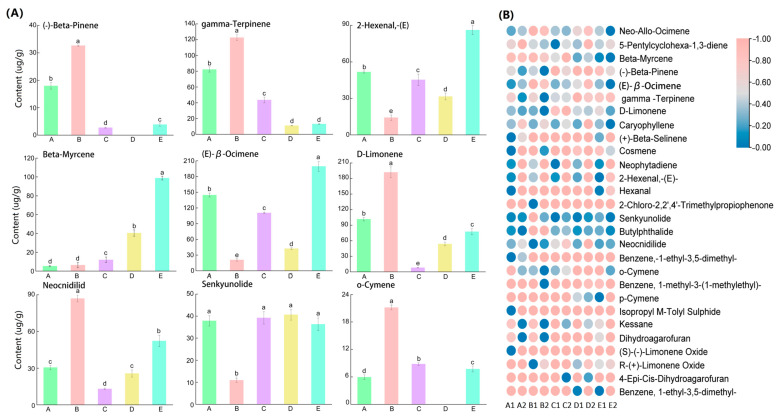
Characteristic compound analysis of high-content compounds (Benzene,-1-methyl-3-(1-methylethyl)-abbreviated as Isopropyl M-Tolyl Sulfide). (**A**) The nine principle compounds in celery. Each error line represents the mean ±SD (standard deviation), *t*-test with significance level of 0.05 (*p* < 0.05). The different letters with lowcase (a–e) indicate significance differences, the same letter indicating no significance difference. (**B**) The correlation between tissues, cultivars, and VOCs. The horizontal axis stands for five cultivars. On the x-axis, from A to E, there were five types of celery. A for ‘Hanyu Nencuixiqin’, B for ‘Jingpin Saixue’, C for ‘Ziyu Xiangqin’, D for ‘Shanghai Huangxinqin’, and E for ‘Siji Lvxiangqin’; 1 for leaves, 2 for petioles.

Twenty-eight characteristic compounds, including eleven terpenes, two polymeric aldehydes, one aldehyde, three esters, six aromatic compounds, and five heterocyclic compounds, were screened to further analyze the contribution of different aroma components to celery. Terpenes and polymeric aldehydes were significantly different in 10 samples, indicating that terpenes and esters were both more abundant in celery than heterocyclic and aromatic compounds. Figure 3 depicts the quantitative composition of VOCs in five celery varieties. Comparing and analyzing nine compounds revealed significant differences in content among the five celery varieties, with most of the compounds showing high levels in *A. graveolens* ‘Jingpin Saixue’ and ‘Siji Lvxiangqin’, such as (-)-β-pinene, γ-terpinene, d-limonene, neocnidilide, and o-cymene in ‘Jingpin Saixue’, 2-hexenal,-(E)-, β-myrcene, and (E)-β-ocimene in ‘Siji Lvxiangqin’; however, senkyunolide was present in all celery varieties with more than 10 µg/g.

**Figure 4:**
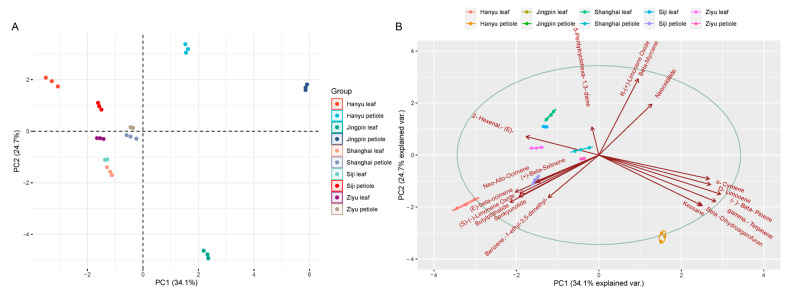
PCA analysis of two tissues, the leaf and petiole, of five celery varieties. (**A**)The summary of PC1 and PC2 and significant variability between different celery varieties and different celery parts. (**B**) Principle analysis of the influence of compounds in different samples.

To investigate the aroma components in different parts of different celery varieties, 18 VOCs from 10 celery samples were selected for PCA and OPLS-DA analysis. PCA analysis revealed that PC1 (34.1%), PC2 (24.7%), and the sum of PC1 and PC2 were 58.8%, with good dispersion, indicating significant variability between different celery varieties and different celery parts (Figure 4A). The compounds most likely to influence compound content were R-(+)-limonene oxide, β-myrcene, (−)-β-pinene, γ-terpinene, and β-dihydroagarofuran, indicating that differences in the content of these five compounds led to differences in terpenoids between sites and different varieties of celery (Figure 4B). 

**Figure 5:**
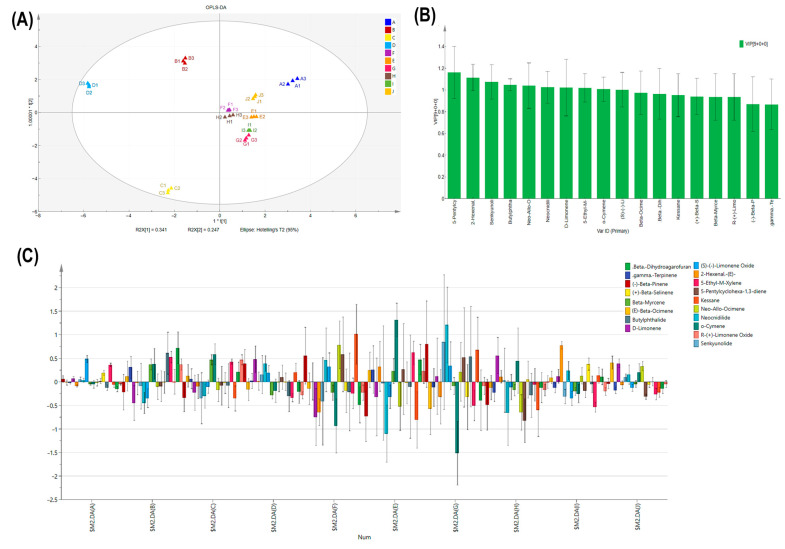
OPLS-DA chart of 18 compounds in five celery varieties. (**A**) Orthogonal partial least squares discriminant analysis of 18 compounds. The score t1 (first component) explains the largest variation of the X space, followed by t2. The numbers besides t[1] and t[2] shown the weight of eh regrression coefficient. (**B**) Variable importance for the projection numbers. (**C**) All compounds differ between two tissues in five celery varieties. The letters (A–J) shows the ten examples of celery, for leaf and petiole of ‘Hanyu Nencuixiqin’, ‘Jingpin Saixue’, ‘Ziyu Xiangqin’, ‘Shanghai Huangxinqin’, ‘Siji Lvxiangqin’, respectively.

An OPLS-DA model was constructed (Figure 5A) with the independent variable fit index R2X = 0.995, the dependent variable fit index R2Y = 0.948, and the model prediction index Q2 = 0.876, with R2 and Q2 exceeding 0.5 indicating acceptable model fit results [31]. Substitution test yielded by a substitution test model of R2 = (0.0, 0.217), Q2 = (0.0, −0.73), and the intersection of the Q2 regression line with the vertical axis was less than 0, indicating that the discriminant model had good explanatory power and the model validation was effective with no overfitting (Figure S1). The aroma of celery varieties and parts can be identified from the results. Variable importance for the projection (VIP) plot (Figure 5B) ranked the top 10 compounds in descending order of importance for the X variable, including 5-pentylcyclohexa-1,3-diene, 2-hexenal,-(E)-, senkyunolide butylphthalide, neocnidilide, d-limonene, 5-ethyl-m-xylene, o-cymene, and (S)-(-)-limonene oxide. There were no variables with VIP values below 0.5, so each of the 18 compounds made a significant contribution. To make the coefficients comparable when the Y variables had different ranges (Figure 5C), the coefficients were normalized (coefficients were divided by the standard deviation of their respective Y variables). The coefficients overview plot revealed that (S)-(-)-limonene oxide was a positive contributor to the leaves and β-dihydroagarofuran had a positive influence on the petioles of *A. graveolens* ‘Hanyu Nencuixiqin’. o-Cymene positively influences ‘Jingpin Saixue’ and ‘Ziyu Xiangqin’ leaves but negatively influences ‘Shanghai Huangxinqin’ leaves. (-)-β-pinene and kessane were positive factors for VOCs of ‘Jingpin Saixue’ and ‘Ziyu Xiangqin’ petioles, while 5-pentylcyclohexa-1,3-diene had an important negative influence on the aroma of ‘Shanghai Huangxinqin’ leaf petioles. 2-hexenal,-(E)- had an important positive influence on the aroma substances of ‘Siji Lvxiangqin’ leaves, and (E)-β-ocimene had an important positive influence on the aroma substances of ‘Siji Lvxiangqin’ petioles.

### 2.4. Validation of Key Regulatory Genes in the Terpenoid Synthesis Pathway

**Figure 6:**
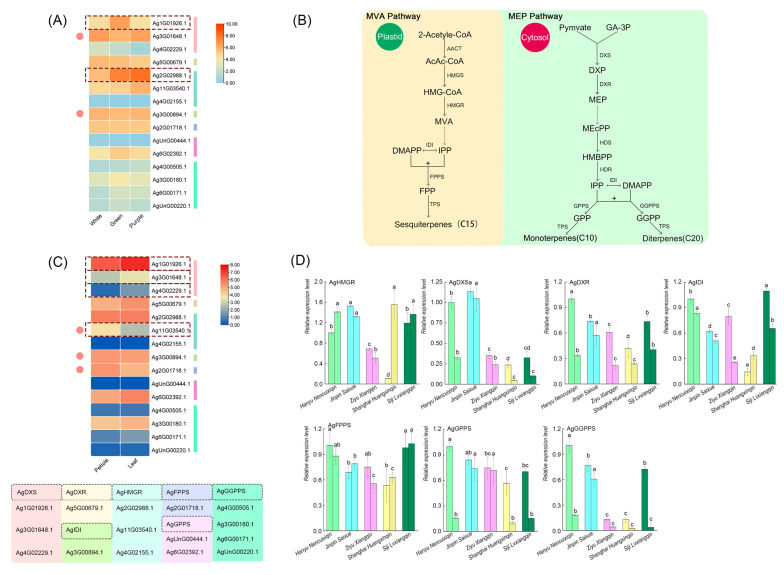
Analysis of key regulatory genes in the terpenoid synthesis pathway. (**A**) Gene expression in distinctively colored celery. (**B**) Key genes and compounds in MVA and MEP pathways. (**C**) Gene expression in distinctive tissues. (**D**) RT-qPCR analysis of gene expression levels in pathway of terpenoid synthesis. Column chart in the same color with the expression of the leaf on the left side and the petiole on the right side. Each error line represents the mean ± SD (standard deviation), *t*-test with significance level of 0.05 (*p* < 0.05). The different letters with lowcase (a–e) indicate significance differences, the same letter indicating no significance difference.

The abundance of genes regulating terpene synthesis in petioles, leaves, and celery varieties (purple, white, and green) was analyzed using published transcriptome data. Fourteen genes with varying levels of transcript abundance were detected in transcriptome samples (Figure 6). *Ag3G01648.1*, *Ag1G01926.1*, *Ag4G02229.1 (AgDXS)*, and *Ag11G03540.1 (AgHMGR)* differ significantly in distinct celery tissues. Gene expression was slightly higher in leaves than in petioles, except for *Ag11G03540.1 (AgHMGR)*, *Ag3G00894.1 (AgIDI),* and *Ag2G01718.1 (AgFPPS)*. All seven genes displayed significant differences between leaf and petiole tissues, indicating that most of the cultivars exhibited higher relative expressions in leaves than in petioles. While different colors of celery express most of the genes at higher levels, *Ag3G01648.1 (AgDXS)* and *Ag3G00894.1 (AgIDI)* are dissimilar. Meanwhile, *Ag1G01926.1 (AgDXS)* expressed significantly higher in green celery, with *Ag2G02988.1 (AgHMGR)* showing the obvious highest expression in purple celery. Based on quantitative validation of five celery leaf and petiole genes, it was determined that the *A. graveolens* ‘Hanyu Nencuixiqin’, ‘Jinpin Saixue’, and ‘Siji Lvxiangqin’ varieties exhibited high expression levels (Figure 6D). Green and white have shown significantly greater expressions than other colors. The heatmaps in Figure 6A,C showed that in the terpenoid synthesis pathway, tissues and petiole color differential expressions were significantly distinct. The white celery, ‘Jinpin Saixue’, had the highest *AgDXS* and *AgHMGR* expressions. Except for *AgHMGR*, ‘Shanghai Huangxinqin’ exhibited the lowest gene expression level. Most of the genes expression, including *AgHMGR*, *AgDXS*, *AgDXR*, *AgIDI,* and *AgGGPPS*, was quite heterogeneous across all five varieties, which reflects the terpenoid synthesis in the MVA and MEP pathways.

## 3. Discussion

GC-MS has been widely used to extract volatile compounds from plants, enabling the separation and detection of numerous VOCs. The combination of stoichiometry for similarities and differences between samples is an important tool for subsequent data analysis due to the large size and complex characteristics of the dataset, such as high variability [32]. In this research, 183 VOCs were detected in the leaves and petioles of the five celery varieties, with terpenes being the most abundant at 60. This is consistent with previous research indicating that the main volatile compounds in celery are terpenes, unlike plants of the Honeysuckle genus, which contain the most compounds as alcohols and acids [30,33]. A total of 18 shared VOCs were detected (neo-allo-ocimene, 5-pentylcyclohexa-1,3-diene, β-myrcene, γ-terpinene, d-limonene, (E)-β-ocimene, caryophyllene, β-ocimene, neophytadiene, nonane, cyclomethicone 5, cyclohexasiloxane, -dodecamethyl-, cyclomethicone 7, naphthalene, neocnidilide, senkyunolide, 2-hexenal,-(E)-, kessane), which was in contrast to other species such as Sanguisorba albanica (isobutyrate, (Z)-4-heptenal) and Jabuticabas (β-cubebene, γ-elemene), and the 18 compounds become differential compounds for celery identification and distinction from other species [34,35]. Data from VOC tables, PCA, and OPLS-DA plots indicated that the composition of compounds in celery was not only related to the color of the petiole and variety but also related to different celery parts that influenced the content and distribution of volatile compounds. Several studies have shown that volatile compounds are positively or negatively correlated with “roasted peanut”, “raw bean”, “dark roast”, and “sweet” attributes, demonstrating the correlation of CIELAB color parameters with roast-related aromas [36]. Green celery had the highest compound content and number of compounds, while white celery had the second highest number of compounds. However, yellow and purple celery had the lowest compound content. Previous research demonstrated that metabolites of different colored petioles were enriched in biosynthetic pathways such as anthocyanins, flavonoids, and the chlorophyll pathway, which influenced the color of celery petioles [37], suggesting that there may be a correlation between the biosynthesis of volatile compound components in celery and the pathway of petiole color formation.

Several genes (*AgTCP3*, *AgTCP4*, etc.) were expressed in all celery tissues during growth and development, with the highest expression of all genes except *AgLEP* and *AgTCP2* found in the leaves, followed by roots, stems, and petioles [38]. Analysis of the compounds based on leaf and petiole content revealed that most celery varieties had higher content in leaves than in petioles; therefore, the differential expression of aromatic compounds in celery in leaves and petioles was related to the differential regulation of celery color and petiole genes.

Terpenes and esters were found in high concentrations in celery, whereas heterocyclic and aromatic compounds were present at low concentrations. Nine of the highlighted compounds differed significantly in content between the five celery varieties, with most showing high levels in *A. graveolens* ‘Ziyu Xiangqin’ and ‘Siji Lvxiangqin’. γ-terpinene, d-limonene, 2-hexenal,-(E)-, and β-myrcene were the most abundant VOCs in celery and could be considered the primary contributors to its aroma. VOCs with VIP values greater than 1 were obtained as follows: 5-Pentylcyclohexa-1,3-diene, 2-hexenal,-(E)-, senkyunolide, butylphthalide, neocnidilide, d-limonene, 5-ethyl-m-xylene, and o-cymene were regarded as potential aroma markers for predicting product quality that can be applied in the processing industry [39].

Terpenoid biosynthesis can occur through the MVA and MEP pathways, with the MVA pathway in the cytoplasm and the MEP pathway in the plastid [40]. This study targeted the expression of key regulatory enzymes of the terpene synthesis pathway to examine the relationship between VOCs, different celery species, and different tissues. Gene expression was slightly higher in leaves than in petioles and significantly higher in green celery, consistent with a previous analysis. Differential gene expressions were significantly distinctive across tissues and petiole color in the terpenoid synthesis pathway. The horticultural plant’s *TPS* gene can regulate the type and content of terpenoids. The MEP pathway is more stoichiometrically efficient than the MVA pathway [41], and genes in the MEP pathway have different relative expressions than those in the MVA pathway, which is consistent with the previous research. All seven genes in the transcription pathway showed significant differences in different tissues of the leaf and petiole, indicating most of the varieties obtained higher relative expressions in the leaves than in the petioles. Expression of kiwi fruit (*Actinidia deliciosa*) sesquiterpene synthases, farnesene (*AdAFS1*), and germacrene synthases (*AdGDS1*) was significantly higher in flowers than in leaf tissue, indicating significant heterogeneity in different plant tissues [42].

In combination with the celery genome data, 15 key genes for terpene synthesis were screened: *DXS*, *DXR,* and *HMGR* genes regulate and control DXP, MEP, and MVA synthesis; *IDI* genes catalyze the isomerization reaction of IPP and DMAPP; and *IDI* genes regulate the terpene raw materials GPP, GGPP, and FPP synthesis of *GPPS*, *GGPPS,* and *FPPS* genes [43]. Genes such as *HMGR*, *DXS*, *IDI*, and *TPS* play a crucial role in terpene production in plants, yeast, and bacteria [44,45]. Most of the celery cultivars exhibited higher relative expressions in leaves than in petioles, with green celery expressing most of the genes at higher levels, and the top three celery varieties with high gene expressions were ‘Hanyu Nencuixiqin’, ‘Jinpin Saixue’, and ‘Siji Lvxiangqin’ varieties exhibited high expression levels. ‘Shanghai Huangxinqin’ exhibited the lowest gene expression level. Yue et al. suggested that the phenylpropanoid biosynthesis and flavonoid biosynthesis pathways could play critical roles during red color fading and aroma formation, which showed that plant color and fragment had a complex correlation [46]. Green celery expressed significantly more than other colors of celery, which was consistent with a previous study that suggested that color groups, like all hydrocarbon monoterpenes, influence terpene synthesis [47]. Previous research by Parmryd et al. discovered that the polypeptide prenylation pattern of the petiole and leaf differed significantly, although some prenylated proteins were present in both tissues and several prenylated polypeptides became more abundant during greening. It is probably about the deep analysis of how different colors of plants show different VOC content [48].

## 4. Materials and Methods

### 4.1. Plant Materials

Five celery varieties were selected as plant materials, including *A. graveolens* ‘Hanyu Nencuixiqin’, ‘Jinpin Saixue’, ‘Ziyu Xiangqin’, ‘Shanghai Huangxinqin’, and ‘Siji Lvxiangqin’, which are widely cultivated in China. The five celery varieties were selected by aroma types and petiole colors. Among them, ‘Hanyu Nencuixiqin’ and ‘Siji Lvxiangqin’ exhibited green petioles. However, ‘Hanyu Nencuixiqin’ is characterized by its tall stature and slightly lighter fragrance, whereas ‘Siji Lvxiangqin’ is shorter and has a stronger flavor. On the other hand, ‘Jingpin Saixue’ has white petioles and a strong scent; ‘Ziyu Xiangqin’ displays purple petioles and an intense flavor; and ‘Shanghai Huangxinqin’ has yellow petioles and a lighter flavor. All celery seeds should be kept for no more than two years. Soak dried celery seeds at 50 °C for 30 min, and then for 24 h at 20–25 °C. Celery seeds were wrapped in moist gauze and placed in an incubator at 20–25 °C for pre-germination, and they were sown when 50% had germinated. Seedling seeds in a nutrient bowl (substrate of nutrient soil: vermiculite: perlite = 3:1:1). When the celery reached commercial maturity (90 days), the petioles and leaves were harvested separately and frozen immediately for later use.

### 4.2. Extraction of VOCs in Celery

For VOC detection, three fresh samples of each of the five celery varieties were snap-frozen in liquid nitrogen and stored in an ultra-low-temperature refrigerator at −80 °C. The extraction was performed by pulverizing the sample in liquid nitrogen and taking 1.0 g of it in a 20 mL glass vial (Agilent Technologies Inc., Santa Clara, CA, USA). In order to inhibit enzymatic degradation, a 5 mL solution of saturated sodium chloride was employed to fully immerse the sample. Furthermore, 10 µL of internal standard 2-octanol was introduced, and the vial was promptly sealed using a polytetrafluoroethylene butyl synthetic rubber spacer. The VOCs were then extracted by stirring at a temperature of 60 °C. A 2 cm DVB/CAR/PMDS SPME fiber (50/30 µm, Supelco Inc., Bellefonte, PA, USA) was exposed to the glass vial headspace for 30 min to enrich VOCs. For GC-MS analysis, the solid-phase microextraction fiber was stretched into the GC system inlet and resolved at 250 °C for five minutes.

### 4.3. Detection of VOCs by GC-MS Analysis Conditions

An extracted sample was introduced into the instrument with a helium carrier gas (99.999% purity) at a forward purge flow rate of 3 mL^.^ min^−1^ and a constant over-column gas flow rate of 1 mL^.^ min^−1^. Agilent DB-WAX (30 m × 250 µm × 0.25 µm, Agilent Technologies Inc., Santa Clara, CA, USA) column was used for VOC separation through an isothermal splitless mode. The GC column temperature was started at 50 °C and increased by 6 °C min^−1^, ramping up to 230 °C and remaining for 5 min. The temperature settings for the transmission line, ion source, and quadrupole mass detector were 250, 230, and 150 °C, respectively.

MS detection was achieved with an electron impact mode of 70 eV and a mass spectrometer scan range of (Mass/Charge Ratio) *m*/*z* 20 to 550 amu. Electron ionization-mass spectrometry (EI-MS) mode was recorded at an ionization energy of 70 eV. The solvent delay time was 0 min.

### 4.4. Analysis of VOCs and Gene Expressions

#### 4.4.1. Qualitative and Quantitative Analysis of VOCs

Computer searches were conducted to match compound spectra with the NIST14 (US) spectral library. A qualitative analysis of VOCs in the petioles and leaves was obtained through manual map resolution and comparison of their retention index (RIS) with compounds reported in the literature. All components were quantified using the peak area normalization method, while the standard was expressed as the 2-octanol content.

The exported data were screened by the NIST14 spectral library for VOC matching, compiled, and statistically analyzed using Excel 2019. Different varieties of VOCs were analyzed using one-way analysis of variance (ANOVA) with IBM SPSS Statistics 27. Origin was used for VOC characterization and analysis, while TBtools was used to reveal specialty compound content and terpene synthesis gene expression. Venn (http://www.ehbio.com/test/venn/ accessed on 12 March 2023) was used to analyze the compound differences in all five varieties [49]. Principal Component Analysis (PCA) was illustrated by https://www.bioinformatics.com.cn (last accessed on 24 May 2023), an online data analysis and visualization platform. SIMCA 14.1 software performed Orthogonal Projections to Latent Structures Discriminant Analysis (OPLS-DA) on the VOCs.

#### 4.4.2. Gene Expression Studies of Terpenoid Synthesis Pathways

The KEGG database (https://www.genome.jp/kegg/ accessed on 15 March 2023) was used to find out the key genes and compounds in terpene biosynthesis pathway. Transcriptome sequencing data in different colors and tissues of celery were downloaded from the National Genomic Science Data Center (https://ngdc.cncb.ac.cn/ accessed on 20 March 2023). Transcription abundance was calculated for genes using FPKM values.

Total RNA was extracted from celery samples by RNA extraction kit (Ai Weidi Biotechnology Co., Ltd., Shenzhen, China). Venus RT6 cDNA synthesis kit (Beijing Qingdao Biotechnology Co., Ltd., Beijing, China) was used to synthesize cDNA. RT-qPCR was performed using the Bio-Rad real-time PCR system and Bio-Rad CFX Manager. Primer sequence was designed by Primer premier 6.0 and is shown in Table S2. Relative gene expression was normalized by *AgACTIN* as a reference gene and calculated by the 2^−ΔΔCt^ method.

## 5. Conclusions

A total of one hundred and eighty-three volatile organic compounds were detected in leaves and petioles of five celery species using GC-MS, including sixty terpenes, thirty-four aromatic compounds, twenty-one ester, twenty-one alcohols, nineteen alkanes, twelve heterocyclic compounds, ten polymeric aldehydes, and six aldehydes. The results of GC-MS and gene expression analysis showed that leaves contained more compounds than petioles, and most of the celery cultivars exhibited higher relative expressions in leaves than in petioles. Eighteen common compounds were screened to provide a basis and foundation for the identification of celery from other species. Eight high VIP-value compounds were identified, which contributed more to the aroma of celery and may become aroma markers for celery quality prediction. The results of the study provide more comprehensive test results for the identification of celery aroma component substances, further providing reference value for the study of celery chemical properties and resource development and utilization.

## Figures and Tables

**Table 1:** Generality compounds table of volatile organic compounds. Approximate quantities of VOCs identified in celery using HS-SPME-GC-MS in leaves and petioles and the total VOC profiles are shown in Table S1. All 19 compounds were selected according to the standard of 5 µg/g content.

Compounds (µg/g)	Hanyu Nencuixiqin		Jingpin Saixue		Ziyu Xiangqin		Shanghai Huangxinqin		Siji Lvxiangqin		CAS-No.
	Leaf	Petiole	Leaf	Petiole	Leaf	Petiole	Leaf	Petiole	Leaf	Petiole	
neo-allo-ocimene	7.43 ± 1.44 b	5.10 ± 0.97 c	0.63 ± 0.03 e	1.18 ± 0.04 de	5.07 ± 0.70 c	6.04 ± 0.75 bc	2.77 ± 0.32 d	1.36 ± 0.29 de	4.77 ± 1.40 c	12.87 ± 2.14 a	007216-56-0
5-pentylcyclohexa-1,3-diene	3.85 ± 0.62 d	2.39 ± 0.54 e	4.63 ± 0.41 bcd	5.22 ± 0.04 b	7.87 ± 0.66 a	4.53 ± 0.23 cd	5.30 ± 0.68 b	2.54 ± 0.50 e	4.86 ± 0.13 bc	4.03 ± 0.12 cd	056318-84-4
β-myrcene	2.46 ± 1.29 fg	3.06 ± 0.73 fg	2.04 ± 0.66 fg	4.47 ± 2.19 f	10.77 ± 2.41 e	1.39 ± 0.51 g	26.33 ± 3.16 c	14.18 ± 0.26 d	42.36 ± 1.16 b	56.50 ± 0.82 a	000123-35-3
(-)-β-pinene	3.75 ± 0.16 d	14.20 ± 1.04 b	5.39 ± 0.31 c	27.24 ± 0.15 a	-	2.72 ± 0.21 e	-	-	0.68 ± 0.21 f	3.17 ± 0.37 de	018172-67-3
(E)-β-ocimene	92.75 ± 1.66 b	51.85 ± 1.96 de	7.12 ± 0.51 h	13.68 ± 1.88 g	62.85 ± 1.69 c	47.66 ± 2.41 e	32.15 ± 0.56 f	10.22 ± 1.12 g	54.84 ± 5.10 d	144.59 ± 4.30 a	003779-61-1
γ-terpinene	10.19 ± 1.26 de	72.01 ± 1.99 b	11.97 ± 0.89 d	110.60 ± 2.69 a	22.46 ± 2.30 c	21.07 ± 1.30 c	2.89 ± 0.57 g	8.13 ± 0.35 ef	7.49 ± 0.15 f	5.61 ± 0.58 f	000099-85-4
d-limonene	38.49 ± 0.83 d	63.05 ± 2.83 b	51.11 ± 2.73 c	140.76 ± 7.81 a	5.55 ± 0.11 g	2.44 ± 0.40 g	24.15 ± 0.14 e	29.60 ± 3.78 e	17.03 ± 1.66 f	60.48 ± 4.38 b	005989-27-5
caryophyllene	2.04 ± 0.03 c	0.47 ± 0.39 e	2.44 ± 0.21 c	0.93 ± 0.06 de	2.58 ± 0.59 bc	1.31 ± 0.42 d	2.64 ± 0.28 b	-	2.07 ± 0.28 bc	4.46 ± 0.17 a	000087-44-5
neophytadiene	2.64 ± 0.03 b	-	1.36 ± 0.03 c	-	3.46 ± 0.20 a	-	1.89 ± 0.69 c	-	2.71 ± 0.33 b	-	000504-96-1
β-ocimene	-	1.23 ± 0.23 a	0.62 ± 0.08 c	1.12 ± 0.01 a	-	0.96 ± 0.17 ab	0.69 ± 0.31 bc	-	-	0.44 ± 0.03 c	013877-91-3
2-hexenal,-(E)-	51.70 ± 0.88 b	-	14.19 ± 2.21 d	-	45.28 ± 4.75 c	-	31.35 ± 2.89 e	0.19 ± 0.05 f	85.18 ± 3.89 a	1.00 ± 0.19 f	006728-26-3
senkyunolide	20.40 ± 0.95 b	17.46 ± 3.32 bc	-	10.93 ± 0.97 d	26.75 ± 3.67 a	12.45 ± 0.51 d	24.24 ± 0.92 a	16.39 ± 3.20 c	10.87 ± 0.46 d	25.40 ± 0.42 a	063038-10-8
neocnidilide	21.33 ± 1.16 d	9.26 ± 0.77 e	54.36 ± 5.02 a	32.54 ± 2.32 b	8.94 ± 0.76 e	4.14 ± 0.13 g	24.42 ± 2.81 cd	1.26 ± 0.12 g	27.73 ± 0.92 c	24.44 ± 2.80 cd	004567-33-3
nonane	0.15 ± 0.10 c	1.54 ± 0.15 a	0.31 ± 0.08 c	1.55 ± 0.03 a	-	0.79 ± 0.19 b	-	0.94 ± 0.22 b	-	0.84 ± 0.12 b	000111-84-2
cyclomethicone 5	0.38 ± 0.05 cde	0.31 ± 0.02 cde	0.42 ± 0.05 cd	0.24 ± 0.01 de	0.45 ± 0.12 c	0.22 ± 0.01 e	0.45 ± 0.10 c	0.24 ± 0.04 de	1.66 ± 0.28 a	1.06 ± 0.02 b	000541-02-6
cyclohexasiloxane,-dodecamethyl-	0.24 ± 0.11 e	0.54 ± 0.120 bc	0.86 ± 0.03 a	0.64 ± 0.12 bc	0.12 ± 0.02 e	0.28 ± 0.06 de	0.48 ± 0.22 cd	0.28 ± 0.11 de	0.75 ± 0.09 ab	0.58 ± 0.06 bc	000540-97-6
cyclomethicone 7	-	0.23 ± 0.01 bc	0.33 ± 0.04 b	0.23 ± 0.05 bc	-	0.15 ± 0.03 c	0.31 ± 0.05 bc	-	0.55 ± 0.21 a	-	000107-50-6
naphthalene	1.70 ± 0.62 a	-	1.07 ± 0.05 b	-	0.45 ± 0.05 c	-	0.39 ± 0.10 c	-	0.69 ± 0.30 bc	-	000091-20-3
kessane	0.54 ± 0.05 f	5.85 ± 0.13 b	0.35 ± 0.04 f	6.27 ± 0.43 a	-	3.14 ± 0.15 c	-	2.20 ± 0.40 d	1.33 ± 0.07 e	-	003321-66-2

Note: The different letters (a–h) indicate significance differences, the same letter indicating no significance difference. The CAS numbers were the compounds IDs to identify the VOCs, which can be searched on the website (https://pubchem.ncbi.nlm.nih.gov/, accessed on 4 January 2023).

**Table 2:** Analysis of eight types of VOC content (µg/g). Eight types of VOCs were detected by HS-SPME-GC-MS, which showed differences between different varieties and tissues.

Varieties	Hanyu Nencuixiqin		Jingpin Saixue		Ziyu Xiangqin		Shanghai Huangxinqin		Siji Lvxiangqin	
Parts	Leaf	Petiole	Leaf	Petiole	Leaf	Petiole	Leaf	Petiole	Leaf	Petiole
Terpenes	179.19	219.41	92.67	315.09	130.06	90.14	80.95	69.41	146.75	305.68
Alcohols	3.02	3.33	0.95	14.42	0.33	0.77	0.81	0.72	5.75	0.00
Polymeric aldehyde	62.41	0.00	14.96	0.40	47.23	0.00	32.20	0.43	97.50	2.11
Aldehydes	0.43	0.00	4.60	0.00	0.66	0.00	0.45	0.00	0.00	0.36
Ester	49.56	27.25	58.03	46.42	41.18	19.26	56.32	21.25	44.54	56.48
Alkane	4.12	4.83	2.77	2.66	1.86	1.95	2.05	2.80	6.60	5.01
Aromatic compounds	16.10	8.50	9.07	22.23	8.18	3.01	10.32	1.96	16.19	8.19
Heterocyclic compounds	21.80	12.09	16.62	13.83	0.99	6.53	5.68	5.28	7.57	2.04
Unknow	0.00	0.00	0.00	0.28	0.00	0.17	0.25	0.00	0.60	0.69

## Data Availability

Not applicable.

## Supplementary Materials

The following supporting information can be downloaded at: https://www.mdpi.com/article/10.3390/ijms241713343/s1.
