# Supplementary material for: Characterization of Volatile Organic Compounds in Five Celery (Apium graveolens L.) Cultivars with Different Petiole Colors by HS-SPME-GC-MS
Source: Int J Mol Sci. 2023 Aug 28;24(17):13343. doi: 10.3390/ijms241713343 (PMC10488006; doi:10.3390/ijms241713343)
Supplement: Supplementary file 1 [file ijms-24-13343-s001.zip › Table S2.pdf]

Table S2. Primer sequences used in this study.

**Gene name**	**Gene ID**	**Forward primer (5'-3')**	**Reverse primer (5'-3')**
AgDXS	Ag1G01926.1	GCACTTATTCGCAGCCTCACAA	TCAGGAAGGACAATCGGTCTCC
AgDXR	Ag5G00679.1	TCACTGTGGACTCCGCTACTCT	CCAGTTGTGCCAGGACTGATGA
AgHMGR	Ag11G03540.1	TGGTGCTCTTGGTGGCTTCAAT	AGCAGGTTCAGGCAAGCAGATT
AgIDI	Ag3G00894.1	AGCACCTTCTGATGGCAAGTGG	CGAGGGTTCCTTTCCGAGCAT
AgFPPS	Ag2G01718.1	GCTGGACTACAATGTGCCTGGA	GGTTGACCTCTGCGTGTGTGA
AgGPPS	Ag6G02392.1	AGGCATGGCAGCAGGTCAGT	CTCCGCATACAGCAGAGCACTC
AgGGPPS	Ag3G00180.1	TGCTATGTCTCGCCGCCTGT	GACCGCCACATCTTCGCCATAA
