# Supplementary figures and images for: Characterization of Volatile Organic Compounds in Five Celery (Apium graveolens L.) Cultivars with Different Petiole Colors by HS-SPME-GC-MS
Source: Int J Mol Sci. 2023 Aug 28;24(17):13343. doi: 10.3390/ijms241713343 (PMC10488006; doi:10.3390/ijms241713343)

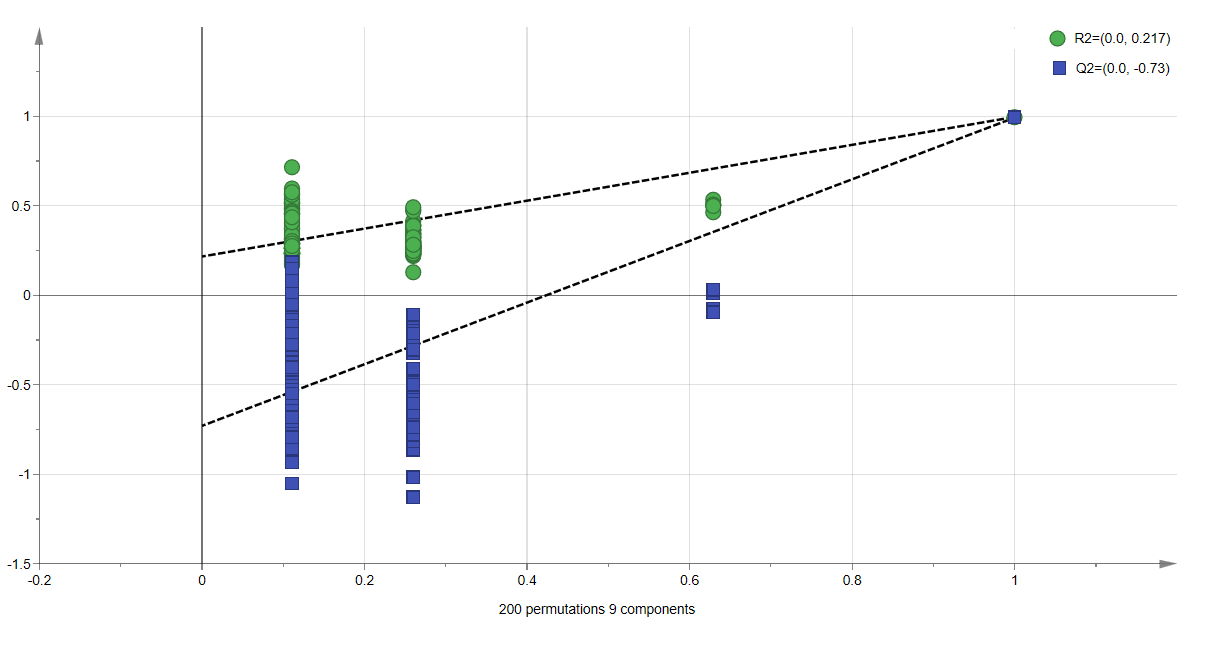

Supplement: Supplementary file 1 [file ijms-24-13343-s001.zip › Figure S1.png]
